# Leveraging Central Sleep Apnea Events to Validate the Measurement of Lung Volume Changes Using Thoracic Bio-Impedance

**DOI:** 10.3390/s26010012

**Published:** 2025-12-19

**Authors:** Martine A. W. Knoops-Borm, Rik Vullings, Hartmut Schneider, Sebastiaan Overeem

**Affiliations:** 1 Department of Electrical Engineering, Eindhoven University of Technology, 5612 AP Eindhoven, The Netherlands; 2 Onera Health, 5617 BD Eindhoven, The Netherlands; 3 American Sleep Clinic, 60389 Frankfurt, Germany; 4 Department of Pulmonary and Critical Care Medicine, Johns Hopkins Bayview Medical Campus, Johns Hopkins University, Baltimore, MD 21218, USA; 5 Kempenhaeghe Centre for Sleep Medicine, 5591 VE Heeze, The Netherlands

**Keywords:** bio-impedance, bioimpedance, bioz, central sleep apnea, thoracic, lung volume

## Abstract

Sleep disordered breathing can cause serious health issues, yet current diagnostic methods are cumbersome and prone to error. Thoracic electrical bio-impedance (BioZ) is a promising alternative, but it remains unclear whether the measured BioZ variations reflect lung volume changes. We leverage linear reductions in lung volume during central sleep apnea (CSA) events to assess whether BioZ measurements capture changes in lungs. BioZ signals from 92 sleep studies were analyzed using linear regression to quantify their slope and linearity ($R^{2}$). Group differences were assessed, and a linear mixed-effects model was used to evaluate the impact of the body mass index (BMI), gender, and sleeping position. Welch’s ANOVA showed significant differences between CSA and breathing segments. A chi-squared analysis showed that CSA events were more likely to exhibit negative slopes. The mixed-effects model found no BMI or gender effects, but the supine posture was significantly associated with more negative linear trends. These findings indicate that BioZ captures lung volume changes and that the sleeping position significantly modulates how clearly these changes appear in the signal.

## 1. Introduction

During sleep, individuals can experience interruptions in breathing whereby little or no air enters the lungs for prolonged periods of time. These breathing pauses are classified by the American Academy of Sleep Medicine (AASM) into different sleep disordered breathing conditions, such as obstructive sleep apnea (OSA) and central sleep apnea (CSA) [1]. These interruptions in breathing cause oxygen levels in the blood to drop to a level that is harmful to the body. The presence of OSA has been associated with hypertension, cardiovascular diseases, and a higher risk of road traffic crashes due to tiredness [2]. OSA and CSA are typically diagnosed based on measurements of breathing effort and airflow during sleep in a clinical or home setting. The current gold standard for measuring breathing effort and airflow into the lungs during sleep is to measure the expansion of the chest using respiratory inductance plethysmography (RIP) belts and air pressure using a nasal cannula. These methods are uncomfortable for the patient and prone to error due to dislodgement [3,4,5]. There is, therefore, a need for a comfortable method of measuring breathing effort and flow that causes minimal disturbance to the patient’s sleep and is reliable throughout the night.

Thoracic bio-impedance (BioZ) is an emerging non-invasive method for estimating respiratory effort and tracking lung volume dynamics [6,7,8,9,10]. It involves the application of a small alternating current across the thorax via surface electrodes, and the resulting voltage is measured to estimate BioZ changes. The region of the thorax within which BioZ changes are sensed using the system is referred to as its sensitivity field. The changes in BioZ reflect fluctuations in this sensitivity field in tissue composition and configuration, similarly to those that take place during the respiratory cycle due to air being drawn into the lungs. Accordingly, BioZ can be used to approximate tidal volume, estimate respiratory effort, and potentially detect abnormal breathing patterns. BioZ pneumography is a promising method for continuous and unobtrusive respiratory monitoring. These characteristics render it suitable for applications such as sleep diagnostics.

Thoracic BioZ is influenced by more than just changes in air volume. Factors such as muscle tone, rib cage expansion, blood flow redistribution, body posture, and cardiogenic oscillations can all impact the BioZ signal [10,11,12,13]. Because all in vivo measurements of BioZ will be confounded by effects outside of air volume changes in the lungs, it is challenging to determine whether changes in BioZ are caused by breathing efforts or by impedance changes in the lung tissue due to changes in air volume. One prerequisite for the BioZ signal to be able to reflect lung volume changes is that the sensitivity field must reach the lung tissue. However, this is rarely validated in practice. Most systems assume that an increase in BioZ during thorax expansion is directly correlated with lung inflation, leaving the precise contributions of airflow and breathing effort to the total BioZ signal largely unverified [10,12,14]. In this study, we aim to determine whether the sensitivity field reaches the lung tissue and verify that BioZ changes indeed reflect changes in lung volume.

CSA episodes are defined by the simultaneous absence of both inspiratory effort and airflow [1]. Previous studies in patients admitted for intensive care have reported a linear downward trend in BioZ during periods of apnea or paused ventilation, which correlated with a gradual decline in residual lung volume over time [15,16]. By only studying the BioZ during CSA events, we can exclude the possibility that impedance changes are caused by breathing efforts and can ascribe these changes to the passive lung volume reduction and, as such, determine whether the BioZ sensitivity field reaches the lungs. Additionally, we investigate which factors contribute to the penetration depth of the sensitivity field into the lungs, such as changes in posture, gender, or body mass index (BMI). Together, these results will indicate whether it is feasible to use tetrapolar thoracic bioimpedance measurements for future respiratory monitoring, particularly for the diagnosis of different forms of sleep disordered breathing, where distinguishing breathing effort from changes in lung volume is essential.

## 2. Materials and Methods

### 2.1. Study Design and Dataset

**Figure 1:**
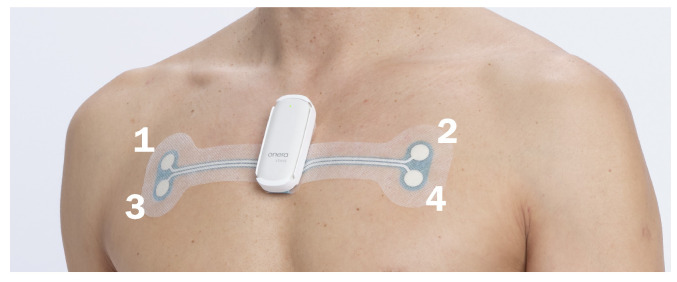
Patch-based tetrapolar device applied on the upper thorax. The superior hydrogel electrodes (indicated by labels 1 and 2) provide an alternating current, whilst the inferior electrodes (indicated by labels 3 and 4) measure the resulting voltage.

**Figure 2:**
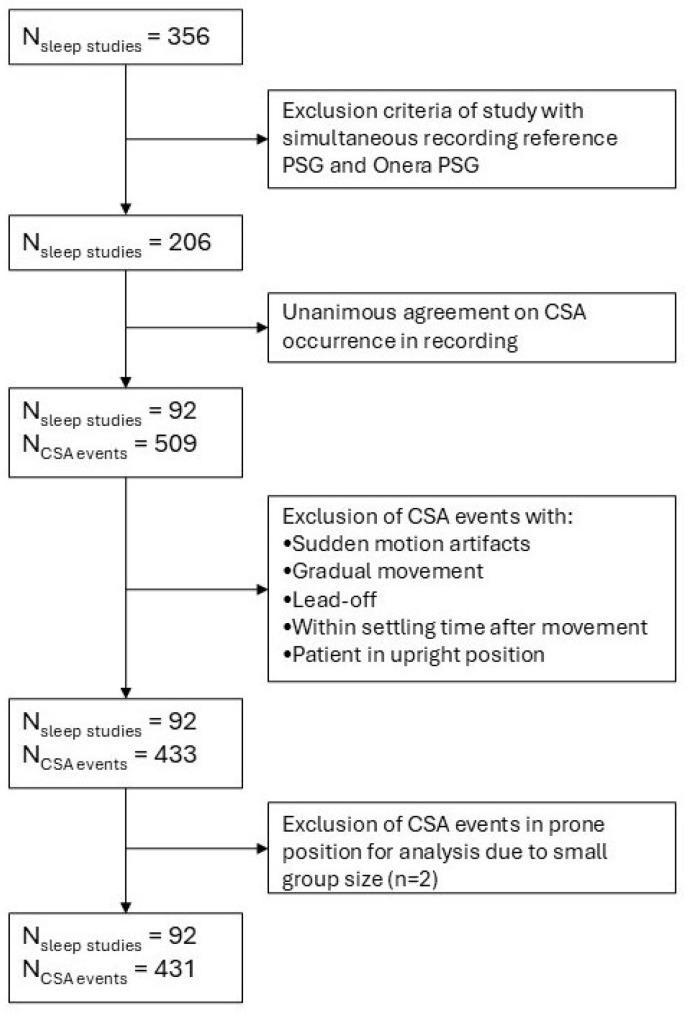
Consort diagram depicting the selection of data for this analysis. Patients referred for a sleep study underwent simultaneous recordings via a reference polysomnography (PSG) system and patch-based tetrapolar device during a single night sleep study [17]. Sleep studies without unanimously agreed upon (by 3 independent sleep scorers) CSA events were excluded from the analysis. CSA events were excluded based on artifact criteria. For the mixed-effects model, a further exclusion was carried out for CSA events that took place whilst the patient was in prone position.

**Figure 3:**
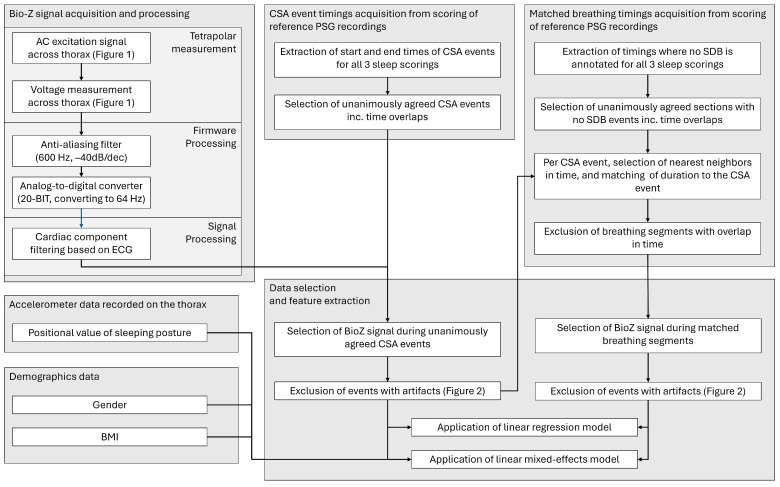
Flowchart describing Bio-Z signal acquisition and processing, the selection of CSA event timings, the selection of matched breathing segments at times of absent sleep disordered breathing (SDB), and the Bio-Z data selection and feature extraction.

Data were collected as part of a multi-center study that compared simultaneous recordings from reference polysomnography (PSG) studies and a patch-based tetrapolar device (Onera B.V., Eindhoven, The Netherlands) in patients referred for a sleep study (Figure 1). The inclusion criteria were such that the patient population represented a wide variety of patients requiring a PSG. In the full dataset of the data collection trial, the overall prevalence of heart disease was 8.3%, hypertension 35.9%, and obstructive lung disease 12.6%. The fully detailed demographics of the original study set can be found in Table 1 of the study of Viniol et al. [17]. The study cohort was recruited from 7 sleep clinics in Germany. Of the 356 recorded studies, 206 passed the general inclusion criteria, and 92 had at least one CSA event scored unanimously in the reference PSG data by three independent sleep scorers. Only unanimously agreed-upon CSA events were used to ensure the inclusion of events with clearly absent effort signals. Scoring on the reference PSG instead of the patch-based sensor was carried out to minimize selection bias in favor of BioZ quality and trends. This resulted in 509 unanimously agreed upon CSA events (Figure 2). The timings of CSA events were used to select BioZ data from the patch-based tetrapolar device (Figure 3).

### 2.2. Bio-Impedance Measurement Device

The tetrapolar bio-impedance measurement device consists of a disposable adhesive patch and a reusable pod containing electronics, a battery, and the system’s memory storage. Contact with the skin was made via four hydrogel electrodes that are integrated in the adhesive patch. An alternating current is injected via the two superior hydrogel electrodes, and the resulting voltage difference is measured via the two inferior electrodes. The sampling rate of the system is 64 Hz (Figure 1).

### 2.3. Signal Processing and Filtering

Using the reference PSG timestamps, the corresponding BioZ signals were extracted from the patch-based tetrapolar recordings. The signals contained several disturbances that can potentially mask trends in the BioZ data. These include cardiogenic oscillations, segments that are too short for detecting gradual trends, edge effects around CSA events (such as gasping for air), signal artifacts due to movement, and spikes due to measurement errors. Therefore, the BioZ signals were preprocessed in the following manner:

- Cardiac component filtering was performed based on the electrocardiogram (ECG) recordings of the patch-based tetrapolar device;
- Segments <10 s were excluded;
- Two seconds were trimmed from the segment edges;
- Segments with sudden motion artifacts (>$10^{\circ }/2$ s) were excluded;
- Segments with gradual movement (>$30^{\circ }$ total) were excluded;
- Segments with lead-off sections (loose electrodes) were excluded;
- Segments with sudden movement before the segment to account for settling time of the BioZ (>$10^{\circ }/2$ s in the 10 s before the start of the segment) were excluded;
- Segments where the patient is in an upright position (position value of $450^{\circ }$) were excluded;
- The interpolation of outliers (>3× standard deviation) was performed.

Accelerometer data from the patch-based tetrapolar device were used for movement filtering. The detection of the lead-off was carried out using the total resistance measured by the patch-based tetrapolar device.

### 2.4. Segment Matching

Segment matching was carried out to increase the comparability of CSA segments and breathing segments both with respect to the total number and the time of occurrence. Each CSA event was paired with two normal breathing segments matched in duration, selected as the nearest valid neighbors in time (before and after the CSA event) for which there was unanimous agreement between the scorers of the no breathing event. Segments were trimmed either at the start (for prior segments) or at the end (for subsequent segments) to align with the CSA duration, ensuring no overlap. If an overlap in time was found between two matched breathing segments, one was removed to avoid duplicate segments. After signal processing and filtering, 92 overnight sleep studies remained, comprising 433 CSA events and 785 matched normal breathing segments.

### 2.5. Feature Extraction

**Figure 4:**
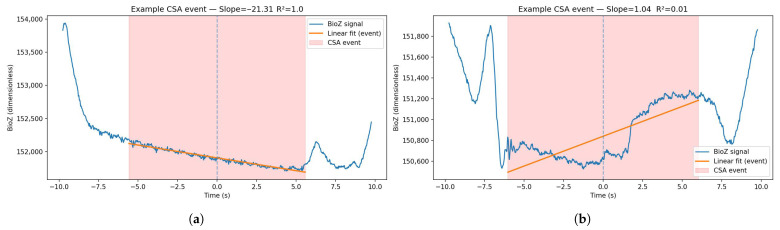
BioZ traces centered around two separate CSA events (highlighted in red) with overlaying linear regression fits: an example of one event with a good fit to the regression and one example of an event where the linear fit does not describe the BioZ trend well. The example events are from two different test subjects. (**a**) Example of the BioZ signal around the time of a CSA event where the linear fit describes the BioZ trend during the CSA event well. (**b**) Example of the BioZ signal around the time of a CSA event where the linear fit does not correctly describe the BioZ trend during the CSA event.

For every CSA and breathing segment, linear regression was performed using linregress from scipy.stats in SciPy (version 1.14.1), calculating two key features:

- Baseline slope of the BioZ signal;
- Linearity of that slope (e.g., $R^{2}$ of linear fit) (Figure 4).

The patch-based tetrapolar device records body position using angular values ranging from $0^{\circ }$ to $360^{\circ }$. These position values were categorized into three bins:

- Prone: $0^{\circ }$–$45^{\circ }$ and $315^{\circ }$–$360^{\circ }$;
- Lateral: $45^{\circ }$–$135^{\circ }$ and $225^{\circ }$–$315^{\circ }$;
- Supine: $135^{\circ }$–$225^{\circ }$.

**Figure 5:**
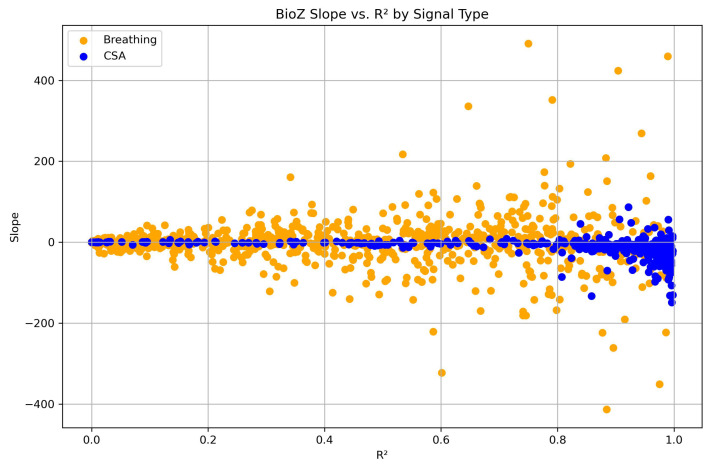
Scatterplot of the slope and linearity ($R^{2}$) of the BioZ signal, as determined from the linear regression fits for each CSA event and the corresponding matched breathing segments used in the analysis.

A total of 312 CSA events occurred in the supine position, 119 in the lateral position, and 2 in the prone position. Due to the limited number of events in the prone position, this category was excluded from the linear mixed-effects model analysis. The linear mixed-effects models were fitted in Python (v. 3.11.11) using the MixedLM implementation from the statsmodels package (version 0.14.4). The spread of the found values for slope and linearity for all data points is visualized in Figure 5.

### 2.6. Statistical Analysis

Levene’s test was used to determine whether there was a significant difference in variance between the CSA and breathing groups for both slope and linearity. Therefore, Welch’s ANOVA was used to compare the differences between the groups in both slope and linearity, as this test is robust to unequal variances. In addition, a chi-square test was performed by categorizing the slope as positive or negative to assess distribution differences between groups. To examine the influence of individual factors on slope and linearity during CSA events, a linear mixed-effects model was applied, with body mass index (BMI), gender, and sleeping position included as fixed effects.

## 3. Results

### 3.1. Group-Level Analysis

Using Welch’s ANOVA revealed differences in linearity ($R^{2}$) between CSA and normal breathing segments, revealing a substantial between-group effect (F = 453.86), with a highly significant difference ($p\approx 1.65\times 10^{-82}$) and a large effect size (partial $\eta^{2}=0.2642$). This indicates that approximately 26.4% of the variance in $R^{2}$ is explained by differences between CSA and breathing. Similarly, when Welch’s ANOVA was applied to slope values, a statistically significant difference between groups ($F=32.48;\;p\approx 1.55\times 10^{-8}$) was observed, but the effect size was small (partial $\eta^{2}=0.0171$), indicating that only 1.7% of the variance in slope can be explained by whether a segment represents CSA or normal breathing. A chi-squared test reflected a significant association between the segment type and a negative slope ($\chi^{2}=110.46;\;p=7.75\times 10^{-26}$). CSA segments were markedly more likely to exhibit negative slopes compared with breathing segments. This trend is confirmed via the contingency table (Table 1), where it can be observed that 82.4% of CSA segments had a negative slope compared with 51.8% of breathing segments. These results indicate that the thoracic BioZ was more likely to exhibit a downward linear trend during CSA events than during normal breathing segments. While treating the slope as a continuous variable showed only a small effect size, treating the slope as a categorical value—whereby we classified the slope as either negative or positive—revealed a statistically significant association with CSA events.

### 3.2. Mixed-Effects Model

**Figure 6:**
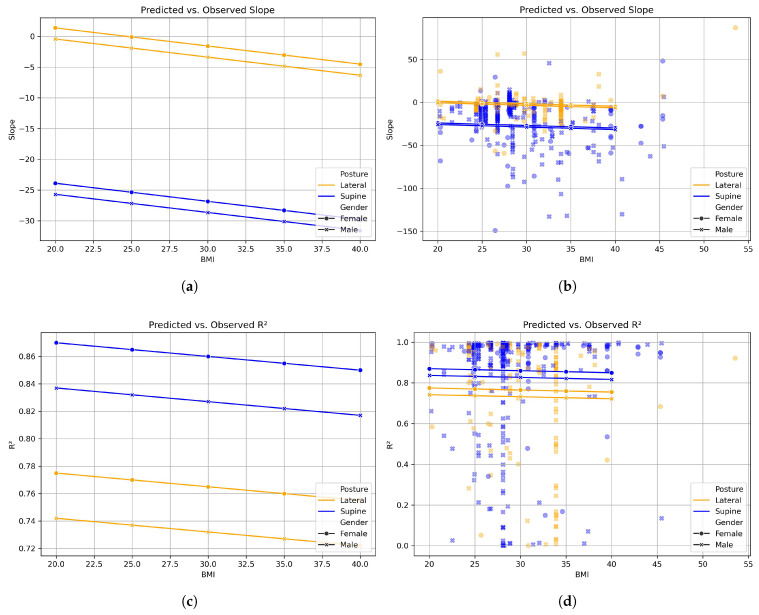
Results of the linear mixed-effects model for the predicted slope and linearity ($R^{2}$) of the BioZ signal during CSA events. The results are differentiated in the male–female and lateral–supine groups. The panels on the left side show the models, and the right side shows the same trend with an additional overlay of the individual data points to visualize the spread of the data. (**a**) Outcome of the linear mixed-effects model for the slope of the BioZ signal during CSA events. (**b**) Outcome of the linear mixed-effects model for the slope of the BioZ signal during CSA events, with an overlay of the data points. (**c**) Outcome of the linear mixed-effects model for the linearity ($R^{2}$) of the BioZ signal during CSA events. (**d**) Outcome of the linear mixed-effects model for the linearity ($R^{2}$) of the BioZ signal during CSA events, with an overlay of the data points.

The linear mixed-effects model of slopes showed that the supine posture was associated with a significant decrease in slope compared with the lateral position (estimate = −25.3, p<0.001). The 95% confidence interval was narrow and did not include zero, indicating a robust and stable effect. Neither BMI nor gender had a statistically significant impact on slope. The model also revealed substantial between-subject variance (group-level variance = 420.9), suggesting that individual differences played a major role in slope variability (Table 2). The model of linearity ($R^{2}$) also showed that the supine posture was significantly associated with higher $R^{2}$ values, indicating greater linearity compared with the lateral position (estimate = +0.095, *p* = 0.005). Gender had a small, non-significant effect (coefficient = −0.033), and the BMI was similarly non-significant with a near-zero coefficient. The group-level variance was low (0.005), suggesting minimal between-subject variability in $R^{2}$ (Table 3). The visualization of the outcome of the mixed-effects model and the spread in the data can be seen in Figure 6. To summarize, the model showed that the supine posture significantly reduces the slope of the BioZ signal during CSA events, independent of gender and BMI. The supine posture was also associated with higher $R^{2}$ values, suggesting that the signal is more linearly stable in this position.

## 4. Discussion

In this study, we confirmed that the sensitivity field of the tetrapolar BioZ device extends sufficiently into the thorax to capture impedance changes occurring in lung tissue. We demonstrated this by showing the presence of a downward linear trend during CSA events, which reflects a progressive decrease in lung volume throughout the apneic episode. Additionally, we showed that the visibility of this decreasing trend in BioZ varies depending on posture, but it is not sensitive to body composition or gender.

The observation of a relatively consistent linear downward trend in thoracic BioZ during CSA events supports the hypothesis that this signal behavior reflects a reproducible physiological process. This trend is aligned with previous reports that the associated BioZ reduces with a decrease in residual lung volume during apnea [15,16]. Therefore, it can be interpreted that these findings show that changes in the thoracic BioZ measured by the patch-based tetrapolar device are at least partially attributable to physiological changes in lung tissue impedance. The discrepancy between the outcome of the ANOVA and the chi-squared test suggests considerable variability in slope values, although the lack of subject-level clustering leaves it unclear whether this reflects intra- or inter-individual differences.

The mixed-effects model showed that the supine position was associated with a more linear and more negative BioZ trend. These findings may reflect physiological and linked signal acquisition differences associated with the body’s position, such as changes in thoracic mechanics, blood flow, or the orientation of sensing electrodes relative to the lungs. When lying on the side, the anterior thorax tends to compress inward, the shoulders rotate forward, thereby repositioning the surface electrodes relatively further away from the lungs. This positional change can, therefore, be expected to reduce the measurement’s sensitivity to BioZ changes in the lungs, diminishing the observed signal.

The finding that BMI and sex did not significantly influence the slope or linearity of the BioZ signal suggests that, at this site of measurement, these demographic factors do not significantly affect the sensitivity field or the system’s ability to detect changes in lung impedance. However, the presence of considerable intra-individual variability in both the slope and linearity across CSA events indicates that other factors may influence the signal on an event-by-event basis. Examples could be changes in blood perfusion between different sleeping positions, electrode–skin contact variability, or an accelerated reduction in lung volume due to catathrenia during a CSA event. Another source of variation could originate from the presence of remaining cardiac oscillations in the BioZ signal, which are one of the primary sources of noise in thoracic BioZ measurements [18]. There were no indications of significant residual cardiac disturbances, but subtle influences cannot be excluded and may have impacted the slope estimates derived from the linear regression analysis, as no post-processing verification was performed after cardiac component filtering. Future research is needed to identify and characterize these contributing factors, with the goal of further refining the interpretability and clinical utility of BioZ signals in sleep and respiratory medicine.

The position of the sensors on the thorax has an influence on the penetration depth of the sensitivity field [14]. In this study, we leveraged a large patient dataset, using signals obtained with an existing product with standardized sensor position. This standardization ensured comparable inter-electrode distances across patients, which was advantageous for the aim of this study but potentially limits the generalizability of the findings to systems using different sensor configurations.

A key advantage of thoracic BioZ measurements is that it is a non-invasive method for assessing respiratory physiology where, unlike techniques such as X-rays, there is no need for exposure to ionizing radiation, rendering it safe for repeated and prolonged use. This method supports continuous, longitudinal monitoring, allowing the detection of trends and dynamics that would be missed in one-time assessments. Additionally, BioZ changes reflect both breathing effort and the air content in the lungs [13]. This renders BioZ particularly well-suited for overnight sleep studies and extended respiratory monitoring both in clinical environments and ambulatory settings. BioZ devices can be of a compact, wireless, and body-worn design that can be discreetly worn under clothing, providing a low-burden and minimally obtrusive solution for patients. This opens up possibilities for home-based monitoring and remote patient management, particularly for individuals with chronic respiratory conditions, such as chronic obstructive pulmonary disease (COPD).

With confirmation that changes in the BioZ signal reflect lung-specific impedance, future research can aim to improve the interpretive accuracy of these measurements. Moreover, by disentangling the lung volume signal components from other sources, future research can carry out more accurate quantitative assessments of respiratory function, resulting in a method that uses BioZ to measure lung airflow and respiratory effort.

## 5. Conclusions

The linearly decreasing baseline was confirmed to consistently characterize CSA events in this dataset. It was shown that sleeping position significantly changes this bio-impedance baseline trend, likely due to changes in the contribution of the lungs to the overall thoracic impedance field of the measurement setup. BMI and sex were found to have no significant influence, supporting the robustness of the signal across a diverse population and indicating that the observed physiological patterns are not significantly affected by body habitus or sex-related anatomical variations. These findings support the use of bio-impedance as a non-invasive marker for characterizing respiratory dynamics.

## Figures and Tables

**Table 1:** Contingency table showing the slope sign of the BioZ signal as determined by the linear regression model for each CSA event and matched breathing segment.

	Positive Slope of BioZ Signal	Negative Slope of BioZ Signal	Total Number of CSA Events/Matched Breathing Segments
Breathing segments	378 (∼48%)	407 (∼52%)	785
CSA events	76 (∼18%)	357 (∼82%)	433

**Table 2:** Mixed linear model regression results: slope of the BioZ signal∼gender + BMI + sleeping posture + (1|sleep study).

Predictor	Coef.	*p*-Value	95% CI
Intercept	7.286	0.626	[−21.999, 36.571]
Gender [Ref. male]	−1.810	0.764	[−13.632, 10.012]
Posture [Ref. supine]	−25.288	<0.001	[−30.761, −19.815]
BMI	−0.295	0.495	[−1.143, 0.552]

**Table 3:** Mixed linear model regression results: $R^{2}$ of the BioZ signal∼gender + BMI + sleeping posture + (1|sleep study).

Predictor	Coef.	*p*-Value	95% CI
Intercept	0.795	0.000	[0.573, 1.017]
Gender [Ref. male]	−0.033	0.453	[−0.118, 0.053]
Posture [Ref. supine]	0.095	0.005	[0.028, 0.161]
BMI	−0.001	0.828	[−0.007, 0.006]

## Data Availability

The data underlying this study are proprietary to Onera B.V. and are not publicly available due to commercial confidentiality and privacy restrictions.

## Abbreviations

The following abbreviations are used in this manuscript:

BioZ	Bio-impedance;
CSA	Central sleep apnea;
SDB	Sleep disordered breathing;
ANOVA	Analysis of variance;
BMI	Body mass index;
AASM	American Academy of Sleep Medicine;
OSA	Obstructive sleep apnea;
PSG	Polysomnography;
ECG	Electrocardiogram;
CI	Confidence interval;
COPD	Chronic obstructive pulmonary disease.
